# Unsteady Foundations: The Effects of Environmental Instability on Nurses and Implications for Nursing Management—An Integrative Review

**DOI:** 10.1155/jonm/9920089

**Published:** 2026-04-21

**Authors:** Ryan Page, Gregory Carter

**Affiliations:** ^1^ School of Nursing, Indiana University Indianapolis, Indianapolis, Indiana, USA

## Abstract

**Aim:**

To determine the state of the science of how instability in the nursing practice environment affects nurses.

**Background:**

The COVID‐19 pandemic, workforce shortages, and an aging population have highlighted the critical need to build and maintain stable nursing practice environments. Factors such as staffing inconsistencies, fluctuating workloads, and workplace violence create instability. While research has explored nursing practice environments broadly, limited research has been conducted on how instability affects nurses.

**Methods:**

An integrative review was conducted using CINAHL, PsycINFO, and PubMed, with search terms related to instability and fluctuations in the nursing practice environment. Articles from 2014 to 2026 were included if they addressed instability in the nursing practice environment and its impact on nurses in hospital settings. Fifteen studies were included in this integrative review.

**Findings:**

Instability in the nursing practice environment has many sources. Organizational support plays a significant role in determining the magnitude and management of environmental instability. Adverse nurse outcomes from instability in the nursing practice environment include decreased well‐being, increased turnover, and workplace violence.

**Implications for Nursing Management:**

Effective leadership and management are necessary to create and maintain positive nursing practice environments, manage environmental instability, and improve nurse well‐being and retention. Targeted strategies such as collaborating with policymakers, strengthening the nursing workforce pipeline, and supporting nurses in their practice environments can mitigate environmental instability and its adverse effects on nurses.

**Conclusions:**

Instability is a common feature of nursing practice environments. Excessive instability can cause adverse nurse outcomes. Nurse leaders are optimally situated to mitigate environmental instability and provide leadership support to improve nurse outcomes.

## 1. Introduction

The COVID‐19 pandemic, workforce shortages, and an aging population have highlighted the critical need to maintain optimal nursing practice environments despite instability in the environment. Since COVID‐19 altered the healthcare landscape, nursing units have been impacted by various destabilizing forces, including high turnover, increased workloads, and greater utilization of travel nurses. Research shows that the quality of nursing practice environments is associated with patient safety outcomes [1]. Creating and maintaining optimal nursing practice environments is crucial for supporting high‐reliability patient care and achieving organizational goals.

Fluctuations in the nursing practice environment may influence the perceived stability of the environment and could affect team members’ and patient outcomes [2]. Instability in the environment may include changes in the number of patients, patient acuity, staffing levels, and resource availability. These conditions can create a perception of uncertainty and make it more difficult for nurses to provide efficient, safe, and compassionate care. The rate, magnitude, and predictability of change in the nursing practice environment may directly influence nurse outcomes and perceptions of the environment.

Although abundant research has been conducted on nursing practice environments [3, 4], the effects of instability in the nursing practice environment have yet to be explored in detail. Current research on nursing practice environments has examined the impacts of the environment on nurse outcomes and associations between healthy work environments, job performance, and productivity [4]. Research has also explored how healthy work environments affect and interact with patient care quality, hospital accidental safety, and nursing leadership [4]. Understanding how instability in the nursing practice environment affects nurses is needed to develop targeted strategies and interventions to support them, improve the work environment, and enable high‐quality patient care. Nurses may experience instability in various ways, such as staffing changes related to staff shortages, changes in policies or practices, and changes in the number, acuity, and workload of patients under their care. However, there is an incomplete understanding of how instability in the nursing practice environment affects outcomes such as nurses’ perception of the workplace, staff well‐being, professional performance, and patient outcomes.

Researchers have used various theoretical, conceptual, and practical lenses to describe instability in the nursing practice environment. The Patient Care Delivery Model (PCDM) based on the Nursing Services Delivery Theory (NSDT) describes instability in the nursing unit environment and how nursing care is delivered [5]. The PCDM and NSDT describe how input, throughput, and output factors interact to influence the nursing practice environment [5, 6]. The NSDT applies Open System Theory concepts [7] to the nursing practice environment [6]. The NSDT describes the nursing practice environment as an energetic system in which entropy in the environment must be counteracted to maintain outputs and organizational existence [6].

Rooted in organizational theory, contingency theory [8] and environmental uncertainty concepts [2, 8, 9] have been used to describe dynamics within the nursing practice environment. Environmental uncertainty, or the perception of uncertainty in the environment, refers to when individuals lack critical information about the environment and cannot predict environmental changes [8, 10]. Environments can be conceptualized as having simple‐complex and stable‐dynamic dimensions [8–12]. Complexity increases as more factors and knowledge are necessary to function [8–12]. The stable‐dynamic dimension is the frequency and predictability of environmental changes [8–12]. This review focuses on the concept of instability within the stable‐dynamic dimension.

External influences such as an aging population [13], healthcare policies [13, 14], technology [15], economic conditions [16], workforce shortages [13], and COVID‐19 [17] have caused instability across the healthcare sector. These external factors may directly shape the nursing practice environment or indirectly influence it through secondary consequences.

The research literature has broadly explored the nursing practice environments, burnout, job satisfaction, and turnover, but research on instability within the nursing practice environment is more limited. Most studies address broader work environment issues, structures, or resources without further exploration of how the unpredictable fluctuations of these inputs into the nursing practice environment affect nurses. It is necessary to fully understand the consequences of instability of inputs into the nursing practice environment to support the nursing workforce and enable high‐quality patient care [18, 19].

Healthcare is constantly changing, highlighting the need to understand the dynamics of the nursing practice environment and its impact on nurses. This literature review aims to determine the state of the science of how instability in the nursing practice environment affects nurses.

## 2. Materials and Methods

### 2.1. Study Design

This study utilized an integrative review methodology to explore how instability in the nursing practice environment affects nurses. This review was guided by Whittemore and Knafl’s [20] integrative review framework and their five‐stage process of problem identification, literature search, data evaluation, data analysis, and presentation. This framework provided a systematic approach that strengthened methodological rigor and enabled a holistic perspective of instability within the nursing practice environment and its effects on nurses [20].

### 2.2. Data Sources and Search Strategy

Articles for review were selected from three databases: CINAHL, PsycINFO, and PubMed. These databases were chosen after consulting with a librarian. Search terms included nurs∗, “environment∗ uncertainty,” “unstable environment∗,” stability, instability, fluctuation, turbulence, unit∗, ward∗, environment, workplace, and hospital.

### 2.3. Eligibility Criteria

Publications were included if the topic addressed instability or changes in the nursing practice environment and its impact on nurses, participants or subjects were registered nurses, the language was English, the article was peer‐reviewed, and the publication date was between 2014 and 2024. The rationale for initially focusing the literature search on the last 10 years was to review the literature pertinent to more modern nursing practice environments. Restricting results further to the post‐COVID literature was preferred; however, the limited research found during this search required a less restrictive approach. To our knowledge, there are no known literature, integrative, or systematic reviews addressing this topic, therefore supporting the use of a broad and inclusive search strategy. Publications were excluded if participants did not include registered nurses, the setting was not a hospital, the study was a literature review, or the study validated an instrument. The CINAHL search was completed on October 8 and 14, 2024. The searches in PsycINFO and PubMed were completed on October 12 and 14, 2024. To update the review for publication, the literature searches in CINAHL, PsycINFO, and PubMed were updated on January 6, 2026, for any articles published since the initial searches.

### 2.4. Study Selection

Articles found in databases during the search phase were exported to Covidence, a web‐based software designed to improve the efficiency of the literature review process. During the screening phase, the primary author was the sole reviewer of articles, utilizing the predefined inclusion and exclusion criteria. The secondary author was involved in the full‐text review to confirm the articles met the study criteria. The reviewers held virtual meetings to discuss articles with any disagreements [21]. After discussing the decision‐making process for each article, consensus was reached on the 15 articles.

The initial search identified 2266 articles, 376 of which were excluded as duplicates. One thousand eight hundred ninety studies were screened, and 1823 were excluded based on inclusion and exclusion criteria by reviewing titles and abstracts. The search found an additional 32 articles after including “turbulence” as a search term, totaling 99 articles. This term was added to the search strategy as it was identified that researchers may utilize “turbulence” as a characteristic of the nursing practice environment. Of these articles, 28 were excluded and 71 were identified for full‐text review. The updated literature search on January 6, 2026, found an additional 413 articles. Of these articles, 411 were excluded and two were identified for full‐text review. Fifty‐eight articles were excluded after full‐text review, and consequently, 15 articles were included for review. Appendix A details the collection process.

### 2.5. Data Extraction

Data from the selected studies were extracted into an Excel spreadsheet. The data extracted included authors, year of publication, journal of publication, title, aim, location, setting, theoretical basis, method, sample, measurement, findings, limitations, and conclusions (Appendix B).

### 2.6. Data Evaluation and Synthesis

While there is no standardized method of article quality evaluation, Whittemore and Knafl [20] suggest customizing it to the types of studies included. JBI’s (formerly known as the Joanna Briggs Institute) critical appraisal tools [22–24] were selected due to their capacity to evaluate studies across a broad range of research designs. Each study was assessed utilizing the relevant JBI checklist to assess methodological quality and risk of bias. These checklists include 6 to 10 questions with response options of yes, no, unclear, and not applicable [22–24]. Both authors completed article quality evaluation independently. After comparing evaluation results, no areas of disagreement or concern were noted. None of the articles had more than two “no” or “unclear” answers, indicating high quality scores. All articles were retained for review.

The articles were imported into NVivo for evaluation and synthesis. NVivo is a qualitative data analysis software that supports coding and thematic analysis across diverse data sources, including journal articles. Thematic analysis was conducted based on Braun and Clarke’s [25] six phases: familiarizing yourself with your data, generating initial codes, searching for themes, reviewing themes, defining and naming themes, and producing the report. After an initial reading, three articles were independently coded, and concepts related to the research question were assigned initial codes. The authors held a coding conference meeting to explore and resolve any differences in coding [26]. Initial codes and interpretations were compared, intercoder agreement was noted, and the remaining articles were coded by the primary author. Codes were iteratively grouped, linked, and constantly compared within and across all articles under review. As patterns emerged, they were identified and documented for analysis, leading to the development of preliminary themes. These themes were then reviewed, refined, defined, and named. The second author assessed the identified codes and themes to verify accuracy and consistency. During two virtual meetings, the reviewers discussed the identified themes, analyzed the findings, and addressed any discrepancies in interpretation. Based on these discussions and analysis, the themes were further refined, reorganized, and consensus was reached among the reviewers.

## 3. Results

The literature synthesis revealed three primary constructs: instability in the nursing practice environment, organizational support, and outcomes associated with environmental instability. These constructs emerged consistently across studies, despite variations in study purpose, methodology, and setting.

Differences between countries exist in healthcare systems, economic conditions, and cultures. Eight studies in this review were located outside the United States [5, 27–33], and six were within the United States [17, 19, 34–37]. The global scope of this review may limit its generalizability to specific countries or local environments.

### 3.1. Study Characteristics

#### 3.1.1. Theoretical Approaches

Three studies were based on the PCDM [5, 27, 28]. Raso et al. [34] used two frameworks: the American Association of Critical‐Care Nurses’ conceptual framework for a healthy work environment and Giordano‐Mulligan’s authentic nurse leadership framework. Miller et al. [35] used the self‐organizing holarchic open systems framework. Ten studies did not list a theoretical basis or conceptual framework [17, 19, 29–33, 36–38].

#### 3.1.2. Study Design and Measurement

Study design and measurement impact the quality of evidence as they affect the validity, reliability, credibility, and generalizability of research findings. Eleven studies used quantitative methodologies [5, 27–32, 34–37]. Among these, seven studies used a cross‐sectional design [27, 30–32, 34, 36, 37], two used descriptive retrospective analysis [28, 29], one used descriptive comparison [5], and one used a prospective observational design [35]. Three studies used qualitative methods [17, 33, 38]. One was a refinement of a concept analysis [38], one utilized an exploratory qualitative design through Zoom interviews [33], and the other used an online form with a pragmatic design [17]. One study used a two‐phase exploratory sequential mixed methods design [19].

Within the 11 quantitative studies, 18 different instruments were used. Eleven of the instruments measured environmental variables, six measured team member characteristics, and one was an instrument that evaluated patient perspectives. Three studies used the Practice Environment Scale (PES) [5, 27, 28], which has five subscales: nurse participation in hospital affairs, nursing foundations for quality of care, nurse manager ability, leadership, and support of nurses, staffing and resource adequacy, and collegial nurse–physician relations [39]. All other instruments in this review were used once.

### 3.2. Instability in the Nursing Practice Environment

#### 3.2.1. Environmental Instability in the Nursing Practice Environment

Nursing practice environments are foundational to healthcare delivery and can impact care quality [5, 31]. These environments are frequently complex [5, 17, 27, 30, 38]. In this context, environmental complexity refers to the scope of factors nurses must evaluate and what knowledge they must use when making decisions [30]. Taken together, these results indicate that nurses must often consider many different factors when prioritizing and deciding how best to deliver care.

When the environment is changing unexpectedly, more information must be considered. Complexity often increases with increased environmental instability [5, 17, 27, 38]. Even shifts with the best staffing may experience environmental instability, such as patient decompensation or nurses getting sick and going home, which can increase nursing unit complexity [5]. Many sources of environmental instability are present in nursing practice environments, contributing to complexity.

Nursing work is often nonlinear and unpredictable [19, 38]. The quantity of environmental instability observed varies based on the timescale in which changes are examined. Over long periods of time, nursing practice environments tend to exhibit more predictable environmental variables such as patient census and staffing [29, 35]. However, day‐to‐day, and even within a shift, nursing practice environments are highly dynamic due to changes in patients, team members, workflow, and resources [5, 17, 19, 29, 30, 35, 38].

Instability in the nurse practice environment can cause disruptions [19, 38]. Turbulence has been described as the disruptive effect on nurses’ workflow caused by thought diversions, resource inadequacy, communication, interpersonal relationships, and workload [19, 38]. Jennings et al. [38] found that the consequences of turbulence include increased cognitive work, workarounds, and diminished well‐being. Overall, these results suggest that environmental instability can cause turbulence [19, 38], which, in turn, can cause adverse nurse outcomes [38]. Table 1 summarizes the effects of instability within the nursing practice environment on nurses, as found in this review.

**TABLE 1:** Effects of instability in the nursing practice environment on nurses.

↑ Complexity [27–29]	↑ Workload [27–29, 33, 37]
↑ Turbulence [19, 38]	↑ Turnover [5, 37]
↑ Cognitive Work [38]	↓ Work Satisfaction [27, 28, 31]
↑ Workarounds [38]	↓ Well‐being [5, 33, 37, 38]

Environmental instability may come from sources external to the direct nurse practice environment. Organizational instability from internal factors such as executive turnover or external factors such as economic conditions and workforce instability can impact nursing practice environments via downstream effects [5, 17, 28, 34, 36, 37]. COVID‐19 was a significant source of external environmental instability, disrupting healthcare and the health of our nursing workforce [17, 33, 36, 37]. Nursing workforce shortages have persisted over time and are a source of instability and complexity in nursing practice environments [17, 28, 34, 36]. External sources may directly contribute to or influence instability in the nursing practice environment.

#### 3.2.2. Nursing Unit Instability

How the literature defines a concept is important, as inconsistent definitions may lead to a lack of consensus on key terms and result in a fragmented body of research. Nursing unit instability has been conceptualized as a characteristic of the local nursing practice environment [5, 28] and as forced involuntary job change [27, 28]. These results indicate a need for researchers to use shared definitions and specific terminology.

Environmental instability within the nursing unit, including changes in patient conditions and team member dynamics, can affect the stability of nursing units [5, 28, 31]. Team members have little control over these environmental changes [5, 30], which may, in turn, cause negative ramifications such as turnover and decreased well‐being [5]. These results show that instability in the nursing practice environment can adversely impact nursing unit teams and individuals.

#### 3.2.3. Team Member Instability

The characteristics of team members have an impact on the nursing practice environment. Instability in team member characteristics, such as increased utilization of contract labor or temporary positions, decreased full‐time employment, team members floating from other units or resource pools, and high absenteeism, contribute to complexity in the nursing practice environment [5, 27, 28, 36]. Declines in team members’ education, experience, and competence may adversely affect the quality and stability of nursing practice environments, as team members with more experience, training, and education may be better able to manage environmental instability [5, 17, 19, 30, 33]. Team members’ experience, education, and abilities may mitigate the impact of environmental instability on nurses.

The composition of nursing teams impacts care delivery. Variations and changes in skill mix, care model, and roles and responsibilities occur in the nursing practice environment [5, 27–29] and may increase environmental complexity and workload for nurses [27–29], particularly if nurses are replaced with support care staff [27]. Appropriate care models are important to delivering efficient, high‐quality nursing care. Specific care models may be more effective at managing instability and mitigating its impact within the nursing practice environment.

### 3.3. Organizational Support

#### 3.3.1. Leadership and Management

Nursing leaders are crucial in improving and sustaining positive nursing practice environments [17, 28, 31]. Strong nursing leadership is associated with decreased intent to leave [27, 28, 31, 34] and greater job satisfaction [28, 31], while concerns with leadership are associated with turnover [17, 36, 37]. Leadership visibility is linked to increased retention [5, 17]. Leaders also play a vital role in the allocation of resources [5, 28, 33]. Positive nursing leadership qualities are associated with a positive nursing practice environment [34] and improved nurse perceptions of care quality [31]. While the importance of good leadership is evident, the perception of the quality of nursing leadership has been predominantly unfavorable [17, 28, 31, 36]. These results highlight the significant influence that leaders have on nurse outcomes.

#### 3.3.2. Staffing and Workload

Appropriate staffing and workload management are key to supporting safe patient care [5, 27, 28, 38]. The literature commonly reports nurse‐reported understaffing [27, 28, 36] and high workloads [17, 28–33, 35–38]. Workload is frequently cited as a reason for turnover [31, 32, 36, 37] and may impact nurses’ ability to provide timely care [17, 27, 28, 30, 38]. Staffing and resource adequacy were rated negatively but have remained stable over time [28, 36]. These findings highlight persistent challenges with appropriate staffing and workload management.

Unpredictable changes in nurse staffing are a source of instability. Staffing and workload varies shift to shift [5, 29, 35, 38], day to day [29, 35], by day of the week [29, 35], seasonally throughout the year [29, 35], and across hospitals [28]. These findings indicate that nursing practice environments are often highly dynamic, causing nurse workload changes (see Table 1).

#### 3.3.3. Positive and Negative Nursing Practice Environments

Nurses’ perceptions of their environments may reflect the quality of the nursing practice environment. Studies across multiple hospitals found that nurses broadly perceived their practice environment negatively [28, 34, 36]. Studies with data at the nursing unit level found some positive practice environments [5, 28]. The perception of a poor nursing practice environment has been associated with adverse patient outcomes such as patient complaints [31], verbal abuse [27, 31], falls [31], nosocomial infections [31], task delays [28], and medication errors [31]. Poor leadership support and staffing have also been associated with perceptions of poor nursing practice environments [27]. These findings reinforce the importance of creating and maintaining supportive nursing practice environments.

Interdisciplinary relationships are key factors that can positively influence the nursing practice environment [17, 28, 31]. Positive teamwork and cultural characteristics can enhance the environment and improve patient and team member outcomes [17, 31]. Strengthening these relationships between team members and improving teamwork are strategies for improving the environment.

### 3.4. Outcomes From Instability in the Nursing Practice Environment

#### 3.4.1. Decreased Well‐Being

Instability in the nursing practice environment may impact nurses’ well‐being (see Table 1). Studies have shown that nurses’ well‐being is at risk [17, 33, 34, 36–38] due to working conditions in the nursing practice environment [17, 28, 32, 34, 37, 38]. Measures of decreased nurse well‐being, such as burnout, emotional exhaustion, and depersonalization, have been associated with higher turnover intentions [17, 31, 36, 37] and adverse patient outcomes [31, 38]. Nursing leadership and a positive work environment are associated with nurse well‐being [34]. Leadership may influence nurse job satisfaction [28, 31, 37] by influencing the nursing practice environment [5, 17, 28, 31, 37]. Nurse work dissatisfaction may be associated with unstable or decreased working conditions [28, 31, 36, 37], similar to well‐being. These findings indicate the importance of managing environmental instability to mitigate potential adverse effects on nurse well‐being.

#### 3.4.2. Workplace Violence

Workplace violence against nurses is a disturbingly common occurrence and has emerged as a theme in the literature. Four studies [27, 31, 32, 36] mentioned workplace violence toward team members, such as physical aggression, threats, and verbal abuse. Experiencing workplace violence is associated with an increased likelihood of turnover [32, 36]. High levels of burnout are associated with patient and family complaints and verbal abuse [31]. Emotional abuse is associated with nursing tasks not being completed [27]. These results show how workplace violence causes wide‐ranging adverse effects on nurses.

Leadership and management play a role in preventing and mitigating workplace violence against nurses. Higher levels of workplace violence may stem from inadequate leadership and organizational support [27] and more severe adverse effects on team members [32]. When workplace prevention policy reaches a certain level, it may offer a protective effect and decrease team members’ intention to leave [32]. Taken together, these results show that leaders have opportunities to develop and implement strategies to mitigate workplace violence and its impact on nurses.

#### 3.4.3. Turnover (Intention)

Retention of nurses is needed to decrease instability in the nursing practice environment. High levels of nurse turnover and turnover intent are reported in the literature [5, 28, 34, 36, 37]. Turnover rates were reported between 15% and 66.9% [5, 37], and turnover intention rates were 13.3%–39.1% [5, 27, 28, 34, 36]. Nurses thought about leaving the profession [17, 31, 34, 36] at rates between 2.5% and 13.1% [31, 34, 36]. Increased workloads [31, 36, 37], insufficient leadership [5, 31, 36, 37], workplace frustration [32], incidents of violence [32, 36], limited nursing experience [27, 36], and concerns over pay and benefits [17, 36] have all been linked to nurse turnover or the intention to leave the profession. These findings show persistent high nurse turnover rates and associated reasons for leaving.

## 4. Discussion

Destabilizing forces affect healthcare and nursing practice environments. Understanding how this instability affects nurses is an important first step to broadly comprehending the nursing practice environment. With this information, healthcare leaders may be better prepared to improve nursing practice environments. This integrative review included thirteen studies, which identified constructs related to instability in the nursing practice environment and examined its effect on nurses. This review is the first to explore the topic of instability in the nursing practice environment. It identified factors contributing to instability in the nursing practice environment, how instability can create adverse nurse outcomes, and how leaders may be able to mitigate instability and improve nursing practice environments. Although many of the identified factors and nurse outcomes are well established in the literature, examining the nursing practice environment through the lens of instability has shown that there are opportunities to further explore, measure, and understand the interaction between nurses and their practice environments.

Previous studies have described the change within an environment as dynamism [8, 10, 12], turbulence [2, 9, 40, 41], and instability [42]. The findings of this review also highlight the need to provide clarity in definitions and phenomena. Although nuances exist, there appears to be some agreement between definitions and usage of dynamism [8, 10, 12] and instability [5, 42]. To align future researchers, the author recommends using instability to describe unpredictable shifts, changes, or fluctuations of inputs into the nursing practice environment. Similarly, turbulence is recommended when describing how environmental instability impacts nurses rather than describing the characteristics of the environment.

The findings of this review suggest that instability in the nursing practice environment often arises from various sources [5, 17, 19, 29, 30, 35, 38], including the external environment, from within the nursing unit, and from individual team members. The external environment can cause instability in the nursing practice environment through factors such as the COVID‐19 pandemic and nursing workforce shortages [17, 33, 34, 36, 37]. The nursing unit may experience chaos when demands exceed the unit’s ability to manage instability effectively. Fluctuations in census, patient acuity, team member turnover, and reliance on non–full‐time staff can cause added complexity and instability in the nursing practice environment [5, 28, 31]. At the team member level, instability can arise from team member characteristics [5, 19, 27, 28, 30, 36] and variations in care model delivery [5, 27–29]. Instability caused by changes in staffing assignments, workload due to patient demands, technology, and breakdowns in communication may result in turbulence [19, 38].

The results of this study indicate that instability in the nursing practice environment has adverse impacts on nurses. Instability disrupts nurse workflows, causing frequent turbulence [19, 38]. The degree of instability in an environment may influence nurses’ perception of a positive practice environment. Poor working conditions, inappropriate staffing, and excessive workloads will likely decrease work satisfaction and well‐being and may lead to turnover. Nurse turnover may create a negative feedback loop by increasing workloads for remaining nurses and compromising the nursing practice environment.

The current study found that organizational support influences instability in the nursing practice environment. Leadership and management may provide instability to the nursing practice environment, or they can help prevent and mitigate it. Organizations and leaders create instability when they fail to clarify or consistently apply policies and procedures. Leaders can minimize environmental instability and increase nursing teams’ ability to handle instability by ensuring appropriate staffing levels and workload distribution. By the nature of the scope of leadership positions, leaders play an influential role in cultivating and maintaining a positive nursing practice environment. They may help shape the culture by setting, communicating, and reinforcing expectations. Positive leadership practices such as communicating effectively, being visible and approachable, recognizing team members, and providing opportunities for growth and development help foster a positive nursing practice environment that promotes nurse well‐being and job satisfaction.

A lack of research examining instability in the nursing practice environment hinders our understanding of how different variables affect the environment and nurses. This gap in research and the lack of validated instruments to measure instability make it difficult to quantify and evaluate its prevalence, characteristics, and impacts. It also makes it difficult to compare findings across studies, assess outcomes from interventions, and develop strategies to address instability in the nursing practice environment.

The absence of findings that support the potential benefits of instability in the nursing practice environment may lead a reader to believe that instability may be inherently harmful. However, researchers must interpret these results with caution because the subject has limited research. Building off prior nursing research [43], uncertainty management theory proposes that the nature of uncertainty is neutral, and emotional responses to uncertainty may be positive, neutral, or negative [44]. Environmental instability may be similar. Nurses’ perceptions of and ability to manage environmental instability may mediate the effects of instability on nurse outcomes.

Albert et al. [45] state that all sustainable and thriving systems respond and adapt to changes in both their internal and external environments. This principle of adaptation is core to the concept of complex adaptive systems (CAS) [46]. Notarnicola et al. [46] describe CAS as dynamic networks of interacting components, like nursing practice environments. These systems self‐organize, adapt to change in the environment, and produce unpredictable emergent outcomes [46]. Nurses continually assess, adapt to, and respond to instability within the nursing practice environment, which in turn influences the environment, creating a complex feedback loop.

Environmental instability exists on a continuum [8–12] where the presence of some instability may build adaptability and resilience in team members and nursing units [47]. Studies suggest that systems achieve their best performance when they adapt well to environments with high levels of instability [48, 49]. Excessive instability can cause chaos in the environment and may cause lasting negative consequences. Too little instability may breed fragility in the system and leave team members unprepared to manage unexpected events [50]. Certain team members may be better able to manage complexity and instability in the nursing practice environment. Similarly, some nursing teams may be better prepared to manage instability. New models of care, technology, and team member development may enable nurses to better manage instability [37].

In this review, most studies did not use a theory, model, or framework to guide the design, implementation, or interpretation [17, 19, 29–33, 36–38]. Researchers found no differences in results when comparing studies with a theoretical basis [5, 27, 28, 34, 35] to those without. Although the studies in this review used appropriate theories and frameworks, the theoretical basis remains limited because researchers need more theories and frameworks explicitly targeting instability in the nursing practice environment. These findings suggest an opportunity to develop a middle‐range nursing unit or nurse practice environment stability theory.

Methods and measurements impact data collection and potential findings. Researchers observed that 11 of the 15 studies in this review used quantitative methodology. While these studies have provided measurable findings that have broad generalizability, the methodological heterogeneity and use of many different instruments limit the synthesis of results. Current instruments have limitations and do not fully capture the complexity and turbulence in nursing work [19] and instability in the environment. The lack of an instrument to measure instability in the nursing practice environment highlights qualitative research’s ability to explore topics of interest with limited prior research.

While 10 studies [17, 19, 29, 30, 33–38] were published after the onset of the COVID‐19 pandemic, only six [17, 33, 34, 36–38] are known to have collected data after the onset of COVID‐19 and only five [17, 33, 34, 36, 37] mentioned COVID‐19 or pandemic effects. Four of these studies were conducted in the United States [17, 34, 36, 37]. Additional contemporary research is needed to assess the impact of instability in the nursing practice environment. The COVID‐19 pandemic has disrupted the nursing workforce and the environments in which nurses practice [17, 33, 34, 36, 37], highlighting the need for further research into its impact on the nursing practice environment, the nursing workforce, and its lasting effects over time.

### 4.1. Implications for Nursing Management

These findings show that leadership and management greatly influence the nursing practice environment [17, 28, 31, 37]. Effective leadership is needed to support nurses at the bedside. The evidence from this study suggests that nurses have perceived poor working conditions over time [28, 34, 36]. Although researchers have noted improvements in recent years [36], leaders must continue focusing on improving nursing practice environments. Organizations can support nurses in managing complexities and instability in the environment by providing leadership and management support, ensuring appropriate staffing and workloads, and cultivating a positive nursing practice environment. Healthcare leaders can mitigate instability in the nursing practice environment by collaborating with policymakers, strengthening the nursing workforce pipeline, and supporting a positive nursing practice environment. These targeted strategies can improve nurse well‐being and retention and support the delivery of high‐reliability, high‐quality patient care.

### 4.2. Limitations

There are several limitations to this review. Because researchers lack consensus on the definition of instability in the nursing practice environment, the chosen search terms may not have captured all available literature, leading to the potential omission of relevant articles. A broader search strategy, including related concepts such as uncertainty or volatility, or the inclusion of additional databases, could have yielded different results. Second, the limited research prevented the inclusion criteria from being narrowed to specific countries or post‐COVID publications, which limited the study’s generalizability. Different findings may result from increased research as differences may be present between countries and pre‐ and post‐COVID. The lack of instruments to measure instability in the nursing practice environment weakens methodological rigor and inhibits the ability to appropriately quantify and analyze the phenomena. Despite rigorous methods, subjective interpretation is inherent to the data synthesis process.

## 5. Conclusion

This integrative review found that instability is frequently present in the nursing practice environment and, when ineffectively managed, leads to adverse effects on nurses, including increased workloads, decreased well‐being, and increased turnover rates. The level of organizational support directly influences the nursing practice environment. Nurse leaders are well positioned to provide needed support and positively influence the culture, operations, and environment of nursing practice. Future research is required to create and validate instruments specific to instability in the nursing practice environment.

## Funding

This research received no specific grant from any funding agency in the public, commercial, or not‐for‐profit sectors.

## Conflicts of Interest

The authors declare no conflicts of interest.

## Figures and Tables

**FIGURE A1:**
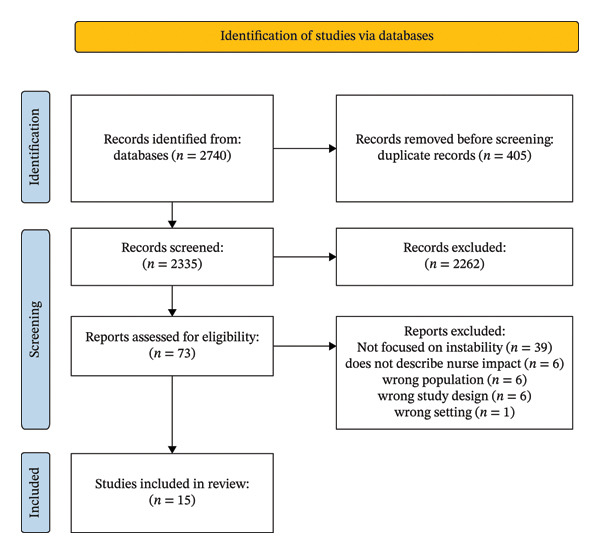
PRISMA flow diagram.

## Data Availability

Data sharing is not applicable to this article as no new data were created or analyzed in this study.

## Appendix B

**Table A1:** Descriptions of reviewed studies table.

Authors (year)	Title	Study aim	Location	Method
Alquwez (2025)	Adapting Nursing Care During the COVID‐19 Pandemic: Staff Nurses’ Experiences, Lessons Learned, and Implications for Nursing Management	To explore the experiences of staff nurses in providing care during the COVID‐19 pandemic and how the nursing care evolved and adapted over time.	Saudi Arabia	Qualitative—exploratory
Alsolami et al. (2023)	Nurses’ Perception of Work‐Environment Uncertainty and Readiness for Organizational Change.	To explore whether there is a relationship between work‐environment uncertainty and nurses’ readiness to participate in organizational change.	Saudi Arabia	Quantitative—cross‐sectional
Browne and Braden (2020)	Nursing Turbulence in Critical Care: Relationships With Nursing Workload and Patient Safety.	The purpose of this research was to specify a definition (of turbulence) and preliminary measure for turbulence.	United States	2‐Phase exploratory sequential mixed‐methods
Chang et al. (2018)	Violence‐prevention climate in the turnover intention of nurses experiencing workplace violence and work frustration.	To determine the moderating effect of a violence‐prevention climate and the mediating effect of work frustration on the relationship between workplace violence (WPV) and the turnover intention of nurses.	Taiwan	Quantitative—cross‐sectional
Church et al. (2025)	Newly‐licensed registered nurses work environment and workforce trends: Analysis of the 2022 National Sample Survey of Registered Nurses	To examine newly licensed registered nurse workforce trends, characteristics, and turnover, comparing 2022 findings with 2018 data to observe post‐pandemic shifts.	United States	Quantitative—cross‐sectional, secondary analysis
Duffield et al. (2015)	Instability in patient and nurse characteristics, unit complexity and patient and system outcomes	To explore key factors related to nursing unit stability, complexity, and patient and system outcomes.	Australia	Quantitative—descriptive comparison
Duffield et al. (2018)	Adding unregulated nursing support workers to ward staffing: Exploration of a natural experiment.	To explore the impact of an initiative to add unregulated nursing support workers to wards in acute care hospitals.	Australia	Quantitative—cross‐sectional
Friese et al. (2024)	Changes in Registered Nurse Employment Plans and Workplace Assessments.	To identify changes in practicing RNs’ employment plans and workplace assessments between the 2022 and 2023 surveys	United States	Quantitative—cross‐sectional
Jennings et al. (2022)	Exploring the turbulent nature of nurses’ workflow.	First, to provide an overview of nurses’ workflow. Second, to examine turbulence by reviewing an early conceptualization, by adding refinements, and by identifying consequences. Third, to address implications for education, the practice environment, and research.	n/a	Concept analysis refinement
Miller et al. (2019)	Using telephone call rates and nurse‐to‐patient ratios as measures of resilient performance under high patient flow conditions	To determine whether commonly collected process measures could provide quantitative evidence for the characteristics of resilient performance in a hospital system.	United States	Quantitative—prospective observational
Musy et al. (2020)	Longitudinal Study of the Variation in Patient Turnover and Patient‐to‐Nurse Ratio: Descriptive Analysis of a Swiss University Hospital.	Aims were to describe the supply and demand dynamics of nursing care and identify mismatches between supply and demand from a longitudinal perspective.	Switzerland	Quantitative—retrospective, descriptive, observational
Raso et al. (2022)	Perceptions of US nurses and nurse leaders on authentic nurse leadership, healthy work environment, intent to leave, and nurse well‐being during a second pandemic year: A cross‐sectional study.	To determine the perceptions of clinical nurses and nurse leaders about authentic nurse leadership, work environment, pandemic impact, well‐being, and intent to leave their position and profession during the second year of the pandemic.	United States	Quantitative—cross‐sectional, descriptive
Roche et al. (2016)	Changes to nurses’ practice environment over time.	To examine changes in the nursing practice environment, retention‐related factors, unit stability, and patient care tasks delayed or left undone, over two periods between 2004 and 2013.	Australia	Quantitative—descriptive, retrospective, secondary analysis
Squires et al. (2022)	Should I stay or should I go? Nurses’ perspectives about working during the Covid‐19 pandemic’s first wave in the United States: A summative content analysis combined with topic modeling.	The purpose of this study was to capture the perspectives of nurses in the United States working the frontlines of the COVID‐19 pandemic’s first wave.	United States	Qualitative
Van Bogaert et al. (2014)	Nursing unit teams matter: Impact of unit‐level nurse practice environment, nurse work characteristics, and burnout on nurse reported job outcomes, and quality of care, and patient adverse events‐‐A cross‐sectional survey.	To investigate the impact of nurse practice environment factors, nurse work characteristics, and burnout on nurse‐reported job outcomes, quality of care, and patient adverse events variables at the nursing unit level.	Belgium	Quantitative—cross‐sectional, survey
